# HIV-1 gp120 induces type-1 programmed cell death through ER stress employing IRE1α, JNK and AP-1 pathway

**DOI:** 10.1038/srep18929

**Published:** 2016-01-07

**Authors:** Ankit Shah, Naveen K. Vaidya, Hari K. Bhat, Anil Kumar

**Affiliations:** 1 Division of Pharmacology and Toxicology, School of Pharmacy, Kansas City, MO 64108; 2 Department of Mathematics and Statistics, University of Missouri-Kansas City, Kansas City, MO 64108.

## Abstract

The ER stress-mediated apoptosis has been implicated in several neurodegenerative diseases; however, its role in HIV/neuroAIDS remains largely unexplored. The present study was undertaken to assess the involvement and detailed mechanism of IRE1α pathway in HIV-1 gp120-mediated ER stress and its possible involvement in cell death. Various signaling molecules for IRE1α pathway were assessed using SVGA cells, primary astrocytes and gp120 transgenic mice, which demonstrated gp120-mediated increase in phosphorylated JNK, XBP-1 and AP-1 leading to upregulation of CHOP. Furthermore, HIV-1 gp120-mediated activation of IRE1α also increased XBP-1 splicing. The functional consequence of gp120-mediated ER stress was determined via assessment of gp120-mediated cell death using PI staining and MTT assay. The gp120-mediated cell death also involved caspase-9/caspase-3-mediated apoptosis. These findings were confirmed with the help of specific siRNA for IRE1α, JNK, AP-1, BiP and CHOP showing significant reduction in gp120-mediated CHOP expression. Additionally, silencing all the intermediates also reduced the gp120-mediated cell death and caspase-9/caspase-3 activation at differential levels. This study provides ER-stress as a novel therapeutic target in the management of gp120-mediated cell death and possibly in the treatment of neuroAIDS.

Despite the advent of combination antiretroviral therapy (cART), the CNS complications associated with HIV-1 infection still present a great challenge in the management of neuroAIDS^1^. These CNS complications, collectively referred to as HIV-associated neurological disorders (HAND), are largely attributed to the BBB disruption, increased pro-inflammatory cytokines/chemokines, increased oxidative stress and neuronal loss^2^. The neurotoxicity of HIV-1 is mainly associated with either the virus itself or the shed viral proteins such as HIV-1 Tat and gp120; however, the exact underlying mechanisms are still unclear^3^. In particular, HIV-1 gp120, the surface glycoprotein which is mainly responsible for viral entry, has previously been shown to increase the CNS toxicity via increase in the pro-inflammatory cytokines/chemokines and oxidative stress in astrocytes and microglia^3^,^4^,^5^,^6^,^7^,^8^.

Endoplasmic reticulum (ER) performs several cellular processes such as synthesis and folding of protein, calcium storage and lipid biosynthesis^9^,^10^,^11^. While several chaperone proteins, oxidizing and glycosylating enzymes and ATP are required to execute these processes, oxidative stress, calcium dysregulation, and lipid overload in the ER lumen^12^ lead to increased unfolded or mis-folded proteins. The accumulation of these unfolded proteins then induce unfolded protein response (UPR) and ER-associated degradation (ERAD)^13^. The UPR is mainly regulated by three major transmembrane proteins that act as stress sensors: inositol requiring kinase I (IRE1), double stranded RNA-activated protein kinase like ER kinase (PERK), and activating transcription factor 6 (ATF6)^14^. Accumulation of unfolded/mis-folded proteins in the ER lumen results in the activation of these signaling molecules to further activate a cascade of downstream proteins^14^. Predominantly, UPR activation is a pro-survival mechanism; however, prolonged activation of these signaling cascades lead to apoptosis^15^,^16^. Along with several other cell death signaling molecules, UPR induces C/EBP homologous protein (CHOP), which leads to apoptotic cell death. Furthermore, the apoptotic cell death is well documented to play an important role in the CNS toxicity of a variety of neurological disorders. However, it is not known whether ER stress-mediated apoptosis plays any role in the CNS toxicity in HIV infected patients.

Several neurodegenerative diseases like Parkinson’s disease (PD), Alzheimer’s disease (AD), Huntington’s disease (HD) and prion related disorders (PrDs) demonstrate accumulation of abnormal protein aggregates in the brain containing specific misfolded proteins^14^,^17^,^18^. Further, HIV-infected individuals were found to produce amyloid beta protein in their brains suggesting a possible involvement of protein mis-folding^19^,^20^. Furthermore, HIV-infected individuals with dementia or Minor cognitive motor dysfunction (MCMD) demonstrate increased grp78/BiP and ATF-6 expression in their brains^21^,^22^. Thus, it is plausible that ER stress plays an important role in the pathology of various neurological disorders including HAND. However, the detailed underlying mechanism(s) is still not clear. More recently, HIV-1 Tat was reported to increase few intermediate molecules of the ER stress signaling pathways in brain microvascular endothelial cell line^23^. However, whether HIV-1 gp120 causes ER stress and if so, its underlying mechanism remains largely unknown.

Although neurons are refractory to the HIV infection, viral proteins are shed from the neighboring astrocytes and microglial cells leading to neuronal loss^24^,^25^. In general, astrocytes serve as a reservoir during the HIV infection since the infection of astrocytes is thought to be restrictive, which allows the virus to enter into latency^26^,^27^. However, recent studies have shown that small population of astrocytes (~5% *in vitro* and 8–10% *in vivo* using an SIV model) can be infected with HIV/SIV^28^,^29^,^30^. In addition, increased astrocyte apoptosis has been reported in the HIV infected patients with severe dementia^31^,^32^. Thus, alteration in the normal physiology of astrocytes can have direct implication on various neurological complications. Therefore, it is important to study the role of HIV-1 gp120 on ER stress-mediated cytotoxicity in astrocytes.

The present study was undertaken to determine whether HIV-1 gp120 induces ER stress and whether it can lead to increased cell death in astrocytes. Furthermore, we also determined the possible role of IRE1 signaling cascade in HIV-1 gp120-mediated apoptosis.

## Results

### HIV-1 gp120 induces the expressions of ER stress markers in time-dependent manner

**Figure 1:**
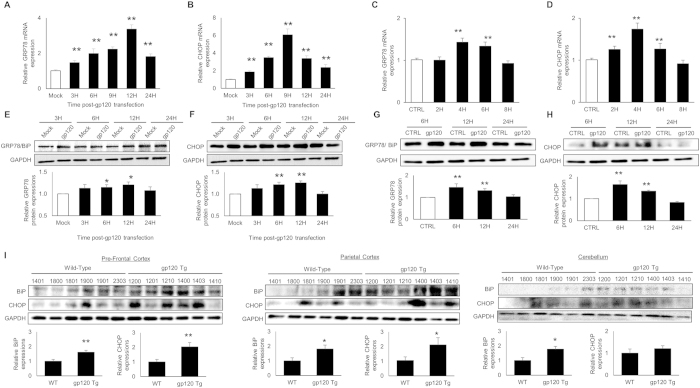
HIV-1 gp120 induces the expressions of GRP78/BiP and CHOP in time-dependent manner. SVGA cells were seeded at 2.5 × 10^5^ cells/well in 12-well plates and transfected using Lipofectamine2000^TM^ with 2 μg plasmid coding for HIV-1 pSyngp120. Cells were harvested at 3, 6, 9, 12 and 24 hours and total RNA was isolated. The expressions of BiP (**A**) and CHOP (**B**) were determined using real time RT-PCR and relative expressions were calculated by comparing controls at respective time. Similarly, the protein levels of BiP (**E**) and CHOP (**F**) were measured at 3, 6, 12 and 24 hours. The relative expressions were calculated with mock-transfected controls at respective time. HFA were seeded at 1 × 10^6^ cells/well in 12-well plates and treated with 200 pM of recombinant HIV-1 gp120 IIIB protein for 2, 4, 6 and 8 hours. The RNA expressions of BiP (**C**) and CHOP (**D**) were measured using real time RT-PCR and the protein levels of BiP (**G**) and CHOP (**H**) were measured at 6, 12 and 24 hours. (**I**) Brains from 4 month-old mice (n = 6 for both WT and gp120 Tg) were collected and protein lysates were prepared. The expressions of BiP and CHOP were measured in PFC, PC and cerebellum using western blotting (**I**). The RNA and protein expressions in all the experiments were normalized with *HPRT* and *GAPDH* as housekeeping genes, respectively. The results with SVGA cells were obtained from at least 3 independent experiments with each performed in triplicates. Similarly, HFA results were obtained from at least 4 different donors. The bar graphs shown in the figure are represented in mean ± S.E., while the western blots are representative images. The blots presented in the figures were obtained by cutting membranes at the molecular markers above and below protein of interest before probing them for appropriate primary and secondary antibodies. The images are then presented as is with brightness/contrast adjustment applied throughout the blot without altering the overall results. Statistical significance was calculated using one-way ANOVA with multiple comparisons and the values were considered significant if p-value ≤ 0.05 (*) or ≤ 0.01 (**).

ER stress is a dynamic process, which involves several intermediate proteins. However, whether this ER stress is pro-survival or pro-apoptotic depends mostly on the duration and extent of the ER stress^15^. Therefore, we measured the levels of key ER stress markers, GRP78/BiP and CHOP after exposure with HIV-1 gp120. We used SVGA cells and transfected them with a plasmid encoding HIV-1 gp120 expression vector for 3, 6, 9, 12 and 24 hours. Total RNA and whole cell lysates were prepared to quantify GRP78/BiP and CHOP at RNA and protein levels. As shown in Fig. 1A,B, increased GRP78/BiP and CHOP mRNA levels were observed as early as 3H post-transfection when compared with control. The peak increase of BiP and CHOP expressions were observed at 12H (3.4 ± 0.2 fold) and 9H (6.1 ± 0.7 fold), respectively. Similarly, the protein levels of BiP and CHOP were measured using western blotting (Fig. 1E,F) and the peak levels of BiP and CHOP were observed at 12H (1.2 ± 0.07 fold and 1.25 ± 0.04 fold, respectively).

To confirm these results in human fetal astrocytes (HFA), we exposed them to 200 pM of HIV-1 gp120 IIIB, a recombinant protein from X4 tropic strain for varying duration of time. Similar to the SVGA cells, the RNA levels of BiP and CHOP in HFA were increased at 4H (1.43 ± 0.10 fold and 1.74 ± 0.15 fold, respectively) as compared to the control (Fig. 1C,D). The protein expressions of BiP and CHOP were also higher in HIV-1 gp120 treated cells as compared to the control, and peak levels were observed at 6H (1.45 ± 0.16 fold for BiP and 1.65 ± 0.16 fold for CHOP) after HIV-1 gp120 exposure (Fig. 1G,H).

In order to confirm the results observed in *in-vitro* studies, we used gp120 transgenic mouse model. The levels of BiP and CHOP were measured in various regions of wildtype (WT) and HIV-1 gp120 transgenic (gp120 Tg) mouse brains. As shown in Fig. 1I, the protein levels of BiP were observed to be higher in prefrontal cortex (PFC) (1.61 ± 0.14 fold), parietal cortex (PC) (1.81 ± 0.27) and cerebellum (1.77 ± 0.20 fold); while the levels of CHOP were found higher in PFC and PC (2.03 ± 0.29 fold and 2.01 ± 0.47 fold, respectively) of gp120 Tg mice when compared to WT control. However, the levels of CHOP in cerebellum were unchanged.

In summary, our results with SVGA cell line, primary HFA and gp120 Tg mice clearly demonstrate HIV-1 gp120-mediated increase in the expressions of BiP and CHOP. In addition, increase in BiP and CHOP were observed to be time-dependent, suggesting that the persistent exposure to the HIV-1 gp120 may result in apoptotic death of astrocytes.

### HIV-1 gp120-mediated ER stress involves activation of IRE1α pathway

**Figure 2:**
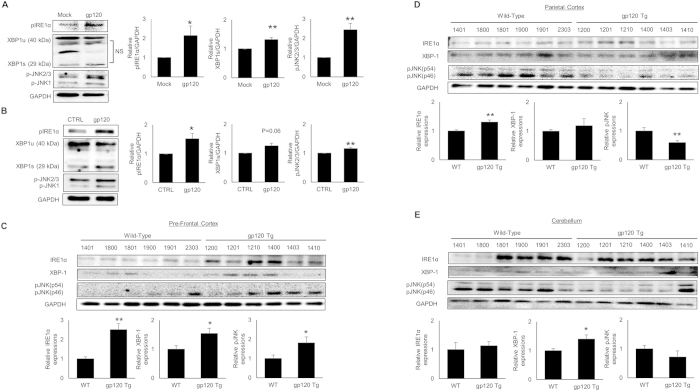
HIV-1 gp120-mediated ER stress involves signaling molecules in IRE1α pathway. SVGA cells were transfected with the plasmid coding for pSyngp120 for 12 hours and levels of IRE1α, phosphorylated JNK and XBP-1 (unspliced and spliced) were measured (**A**). Similarly, primary astrocytes were treated with 200 pM of HIV-1 gp120IIIB for 12 hours and the expressions of IRE1α, phosphorylated JNK and XBP-1 (unspliced and spliced) were measured (**B**). The brain levels of IRE1α, pJNK and XBP-1 in the gp120 Tg and WT mice were measured in PFC (**C**), PC (**D**) and cerebellum (**E**). The results with SVGA cells were obtained from at least 3 independent experiments. Similarly, the HFA results were obtained from at least 4 different donors. For expression levels in the brains of mice, n = 6 each of WT and gp120 Tg mice were used. The bar graphs shown in the figure are represented in mean ± S.E., while the western blots are representative images. The blots presented in the figures were obtained by cutting the membranes at molecular weight markers covering protein of interest before probing them for appropriate primary and secondary antibodies. The images are then presented as is with brightness/contrast adjustment applied throughout the blot without altering the overall results. The statistical significance was calculated using one-way ANOVA with multiple comparisons and the values were considered significant if p-value ≤ 0.05 (*) or ≤ 0.01 (**).

The UPR is initiated upon the release of BiP from ATF6, IRE1α and PERK at the ER membrane, which then activate their downstream signaling cascades^33^. Furthermore, apoptotic cell death under prolonged/severe ER stress has been shown to involve IRE1α and PERK signaling pathways^34^. In the current study, we focused on the involvement of IRE1α pathway in HIV-1 gp120-mediated ER stress. The activation of IRE1α signaling primarily involves two key mechanisms; splicing of XBP-1 mRNA and activation of JNK pathway^35^,^36^. Therefore, we assessed the effect of HIV-1 gp120 on the expressions of IRE1α, phosphorylated JNK and XBP-1 (unspliced and spliced) in SVGA cells (Fig. 2A), primary HFA (Fig. 2B) and gp120 Tg mice (Fig. 2C–E). As shown in Fig. 2, the expressions of all these molecules were increased after gp120 exposure in SVGA cells and HFA astrocytes. In particular, increase in IRE1α, XBP-1s and pJNK expressions in SVGA cells were 2.14 ± 0.5 fold, 1.32 ± 0.07 fold and 2.44 ± 0.34 fold, respectively. Similarly, HFA showed increases in IRE1α, XBP-1s and pJNK expressions by 1.52 ± 0.19 fold, 1.25 ± 0.1 fold and 1.16 ± 0.04 fold, respectively. However, increase in the expression of these proteins in various regions of mouse brain was region specific. In particular, the expressions of IRE1α (2.52 ± 0.30 fold), pJNK (1.81 ± 0.30 fold) and XBP-1 (1.54 ± 0.18 fold) were higher in PFC of gp120 Tg mice when compared to age-matched WT mice (Fig. 2C). However, PC of gp120 Tg mice demonstrated increase in the levels of IRE1α (1.31 ± 0.07 fold), decrease in pJNK (0.6 ± 0.07 fold) and no change in XBP-1 (Fig. 2D). Finally, cerebellum of gp120 Tg mice did not show any changes in the expressions of IRE1α and pJNK but XBP-1 was increased (1.39 ± 0.15) compared to age-matched WT mice (Fig. 2E).

**Figure 3:**
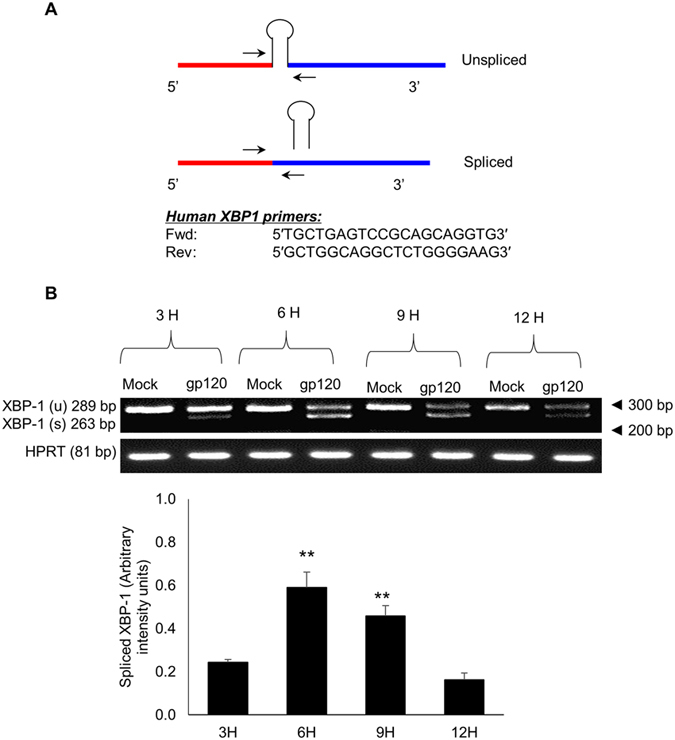
HIV-1 gp120 increases XBP-1 splicing in time-dependent manner. (**A**) The schematic represents XBP-1 RNA processing. The loop represents the 26-nt region being removed during splicing of XBP-1 and theprimers were designed in order to amplify both the spliced and unspliced XBP-1. (**B**) SVGA cells were seeded at 2.5 × 10^5^ cells/well in 12-well plates and transfected with pSyngp120 plasmid for 3, 6, 9 and 12 hours. The RNA were isolated using RNeasy mini kit and XBP-1 RNA were amplified using the primers shown in 3A. The amplified product was resolved using 3.5% agarose gel (**B**). The intensity of the spliced XBP-1 was measured and the mean ± S.E. was calculated from at least 3 experiments. The gel shown here is a representative of 3 independent experiments. The statistical significance was calculated using student’s t-test between 3H and respective time-points to show significant increase over time and the values were considered significant if p-value ≤ 0.01 (**).

As shown in Fig. 3A, XBP-1 mRNA is spliced via removal of 26 nucleotides from unspliced XBP-1 by IRE1α. In order to determine whether HIV-1 gp120-mediated activation of IRE1α played any role in the splicing of XBP-1, we determined spliced and unspliced XBP-1 on agarose gel after HIV-1 gp120 exposure in SVGA astrocytes (Fig. 3B). The peak levels of spliced XBP-1 were observed 6H after transfection.

Altogether, these results clearly indicated that HIV-1 gp120-mediated ER stress involved IRE1α pathway. Furthermore, activation of IRE1α resulted in splicing of XBP-1 and activation of JNK, which then led to increased expressions of CHOP.

### Depletion of IRE1α, JNK and AP-1 using siRNA reduced the expressions of HIV-1 gp120-mediated CHOP

**Figure 4:**
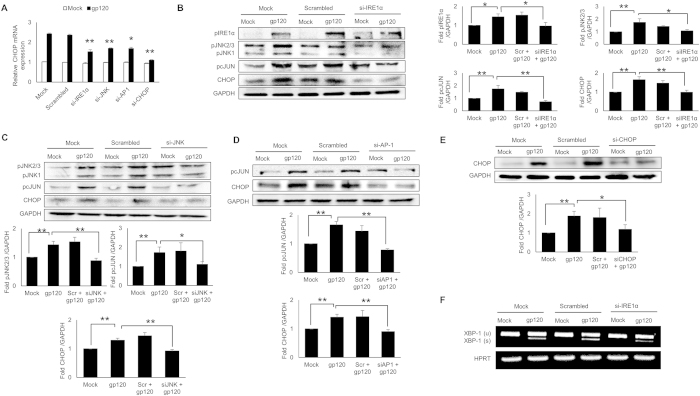
HIV-1 gp120-mediated increase in CHOP expressions is reduced via knockdown with siRNA against IRE1α, JNK, AP-1 and CHOP. SVGA cells were transfected with siRNA against IRE1α, JNK, AP-1 or CHOP for 48 hours as described in methods followed by transfection with plasmid coding pSyngp120 for 9 hours (RNA) and 12 hours (protein). (**A**) The levels of CHOP RNA was measured following depletion of either IRE1α, AP-1, JNK or CHOP using their respective siRNA. (**B–E**) The levels of CHOP protein were measured after depletion of IRE1α, JNK, AP-1 or CHOP using their respective siRNA. The levels of JNK and pcJUN were assessed as a result of IRE1α knockdown (**B**). The levels of pcJUN was measured upon knockdown of JNK to establish link between JNK and AP-1 (**C**). The levels of pcJUN and CHOP were measured upon knockdown of AP-1 to establish the link between AP-1 and CHOP (**D**). The effect of CHOP knockdown was measured on gp120-mediated CHOP expression (**E**). The effect of IRE1α knockdown was assessed on XBP-1 splicing by detection of spliced and full variants of XBP-1 using agarose gel as described in methods (**F**). The results with SVGA cells were obtained from at least 3 independent experiments. The bar graphs shown in the figure are represented in mean ± S.E., while the western blots are representative images. The blots presented in the figures were obtained by cutting the membranes at molecular weight markers covering protein of interest before probing them for appropriate primary and secondary antibodies. The images are then presented as is with brightness/contrast adjustment applied throughout the blot without altering the overall results. The statistical significance was calculated using one-way ANOVA with multiple comparisons and the values were considered significant if p-value ≤ 0.05 (*) or ≤ 0.01 (**).

In order to confirm involvement of IRE1α pathway in HIV-1 gp120-mediated ER stress, we determined CHOP at RNA and protein levels after depletion of IRE1α signaling molecules. Briefly, IRE1α, CHOP, JNK and AP-1 were silenced using siRNA followed by transfection with HIV-1 gp120 in SVGA cells. The levels of CHOP mRNA and protein were determined after 9H and 12H, respectively. The knockdown efficiency of all siRNA was found to be 30–60% (Supplementary Fig. 1). As shown in Fig. 4A, level of CHOP RNA was reduced after IRE1α, CHOP, JNK or AP-1 silencing at variable extents. These results were confirmed at protein levels (Fig. 4B–E). In order to confirm that HIV-1 gp120-mediated activation of IRE1α led to activation of JNK and its transcription factor AP-1, we determined the effect of IRE1α knockdown on phosphorylated JNK and cJUN (Fig. 4B). Similarly, JNK-mediated activation of AP-1 was determined by measuring phosphorylated cJUN levels in JNK depleted cells (Fig. 4C). The effect of AP-1 knockdown on CHOP expression finally demonstrated the link between AP-1 and CHOP (Fig. 4D). The effect of CHOP knockdown was assessed on CHOP expressions (Fig. 4E). Finally, in order to confirm the involvement of IRE1α in XBP-1 splicing, we assessed the XBP-1 splicing using agarose gel in IRE1α depleted astrocytes (Fig. 4F). Altogether, these results further confirmed that HIV-1 gp120-mediated ER stress involved IRE1α and its downstream signaling cascades since knockdown of these genes not only reduced the expressions of CHOP but also their intermediates linking them to CHOP.

### HIV-1 gp120-mediated ER stress increased cell death in astrocytes

**Figure 5:**
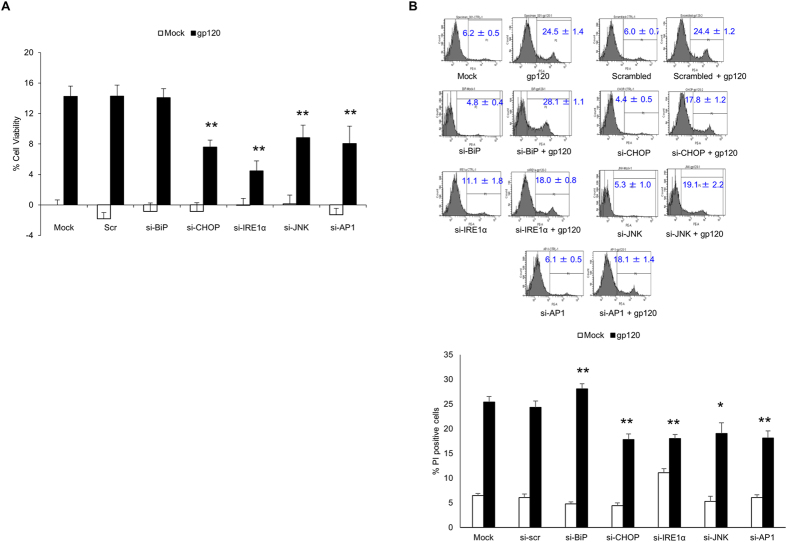
HIV-1 gp120-mediated ER stress increased cell death in astrocytes. Cell death was measured at 48 hours after the transfection of SVGA cells with plasmid for HIV-1 gp120. Briefly, SVGA cells were seeded at 1 × 10^6^ cells in a 6-well plate and transfected with siRNA for molecular intermediates in the IRE1α pathway as mentioned methods. After 48 hours, the cells were reseeded in 12-well plate and allowed to adhere overnight. Finally, these cells were transfected with either mock or gp120 plasmid for an additional 48 H and MTT assay was performed to assess the effect of IRE1α, BiP, CHOP, AP-1 or JNK knockdown (**A**). The % cell death was calculated considering the absorbance in mock-transfected control as 100% viability. (**B**) Similarly, to confirm these results, PI staining was performed at 48 hours post-transfection with HIV-1 gp120. The PI staining was measured by flow cytometry analysis as mentioned in methods. The histograms shown are representative of at least three independent experiments with each performed in triplicates. The results are reported in mean ± S.E. of % cell death calculated with % cells in the P1 gated region. The statistical significance was calculated using one-way ANOVA with multiple comparisons and the values were considered significant if p-value ≤ 0.05 (*) or ≤ 0.01 (**).

In order to determine the functional implication of HIV-1 gp120-mediated ER stress, we assessed the effect of HIV-1 gp120 on cell death via MTT assay and PI staining. Briefly, astrocytes transfected with HIV-1 gp120, showed 17.3 ± 1.7% cell death (Fig. 5A). Whereas, silencing IRE1α, CHOP, AP-1 and JNK with siRNA reduced the HIV-1 gp120-mediated cell death at various extents (4.1 ± 1.7% to 8.8 ± 2.1% Vs 17.3 ± 1.7% in HIV-1 gp120 transfected cells) except BiP (14.1 ± 1.2%). Similarly, cell death observed in PI staining was observed to be 24.5 ± 1.4% in HIV-1 gp120 as opposed to 6.2 ± 0.5% in mock transfected control (Fig. 5B). The depletion of IRE1α, CHOP, JNK and AP-1 via siRNA resulted in reduction of cell death (18.0 ± 0.8%, 17.8 ± 1.2, 19.1 ± 2.2% and 18.1 ± 1.4%, respectively). In contrast, knockdown of BiP slightly increased HIV-1 gp120-mediated cell death (28.1 ± 1.1% as opposed to 24.5 ± 1.4%). These results clearly demonstrated that HIV-1 gp120-mediated ER stress resulted in cell death in astrocytes. Furthermore, depletion of various signaling molecules in the IRE1α pathway reduced HIV-1 gp120-mediated cell death differentially.

### HIV-1 gp120-mediated cell death involved caspase-3 and caspase-9

**Figure 6:**
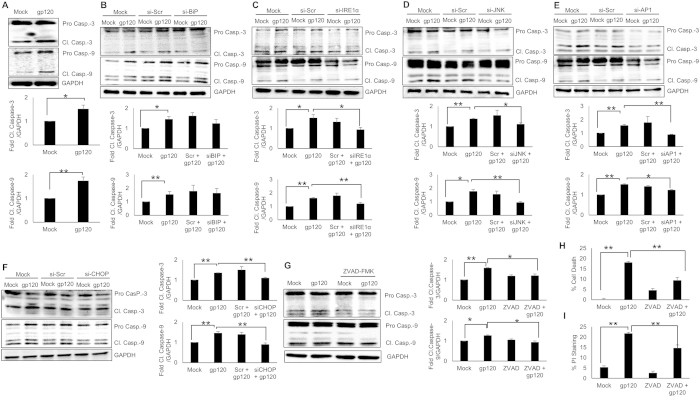
HIV-1 gp120-mediated ER stress involved caspase-3 and caspase-9 in astrocyte cell death. (**A**) The levels of both cleaved and full caspase-3 and caspase-9 were detected in total cell lysates obtained from the SVGA cells transfected with HIV-1 gp120 for 24 hours. (**B–F**) The expressions of IRE1α, JNK, AP-1 and CHOP were knocked down using specific siRNA followed by transfection with HIV-1 gp120 for 24 hours and the levels of cleaved and pro-caspase-3 and caspase-9 were measured. The effect of BiP (**B**), IRE1α (**C**), JNK (**D**), AP-1 (**E**) and CHOP (**F**) knockdown was determined using western blotting. The effect of pan-caspase inhibitor, ZVAD-FMK (20 μM) was assessed on cleaved and pro caspase-3 and caspase-9 (**G**). Similarly, its effect on cell death was assessed using MTT assay (**H**) and PI staining (**I**) at 48 hours after gp120 transfection as mentioned in methods. The bar graphs shown in the figure are represented in mean ± S.E., while the western blots are representative images. The blots presented in the figures were obtained by cutting the membranes at molecular weight markers covering protein of interest before probing them for appropriate primary and secondary antibodies. The images are then presented as is with brightness/contrast adjustment applied throughout the blot without altering the overall results. The statistical significance was calculated using one-way ANOVA with multiple comparisons and the values were considered significant if p-value ≤ 0.05 (*) or ≤ 0.01 (**).

Having demonstrated that HIV-1 gp120-mediated ER stress resulted into cell death in the astrocytes, we wished to determine the underlying mechanism for cell death. Since various signaling molecules in the caspase cascade are classically known to be involved in the apoptotic cell death, we assessed the levels of different caspases. Particularly, caspase-3 and caspase-9 were found to be increased as a result of HIV-1 gp120 exposure (Fig. 6A). Since these caspases are known to get converted into cleaved forms in the event of apoptosis, we focused on the cleaved caspase-3 & 9. Similar to procaspase-3 and procaspase-9, HIV-1 gp120 increased the expressions of cleaved caspase-3 and cleaved caspase-9 by 1.51 ± 0.17 and 1.73 ± 0.13 fold, respectively in SVGA cells. In order to assess the role of IRE1α in HIV-1 gp120-mediated apoptosis, we employed various siRNA against BiP, IRE1α, JNK, AP-1 and CHOP. Clearly, knocking down the expressions of IRE1α, JNK, AP-1 and CHOP using siRNA reduced HIV-1 gp120-mediated cleaved caspase-3 and cleaved caspase-9 expressions (Fig. 6C–F). It was noteworthy that knockdown of BiP did not reduce HIV-1 gp120-mediated cleaved caspase-3 & 9. This was in accordance with our observations in MTT and PI staining, where knockdown of BiP did not prevent HIV-1 gp120-mediated cell death (Fig. 5). To confirm that caspase-3 and caspase-9 were indeed involved in HIV-1 gp120-mediated apoptosis, we employed ZVAD-FMK, a pan-caspase inhibitor peptide. Clearly, SVGA cells pretreated with ZVAD-FMK showed significantly less cleavage of caspase-3 and caspase-9 (Fig. 6G). Additionally, when assessed using MTT and PI staining, ZVAD-FMK significantly reduced HIV-1 gp120-mediated cell death (Fig. 6H,I). To our surprise, ZVAD-FMK reversed only partial effect of HIV-1 gp120. This may be attributable to involvement of caspase-independent cell death mechanisms such as AIF-mediated apoptosis or autophagy^37^ in HIV-1 gp120-mediated cell death, which requires further investigation. Together, these results suggested that increased expressions of cleaved caspase-3 and caspase-9 due to HIV-1 gp120-mediated ER stress led to apoptotic cell death in astrocytes and this increase involved IRE1α signaling pathway.

## Discussion

During the post-HAART era, the incidences of HIV dementia have significantly reduced; however, MCMD and other HIV associated neurocognitive disorders continue to affect a large population of HIV infected individuals^1^,^2^. The CNS toxicity among these patients is largely attributed to various viral proteins including HIV-1 Tat, nef, vpr and gp120^38^,^39^,^40^. In particular, several studies including ours have reported the role of HIV-1 gp120 in CNS toxicity, which involves increase in pro-inflammatory cytokines/chemokines, increased oxidative stress and altered BBB integrity and calcium homeostasis^3^,^4^,^5^,^6^,^7^,^41^,^42^. Our findings in current report for the first time demonstrate that HIV-1 gp120-mediated astrocytic apoptosis involves ER stress. Furthermore, increase in ER stress, as indicated by increase in the levels of BiP and CHOP, also activated caspase cascade in this process leading to cell death. We also employed siRNA against various molecules in the IRE1α pathway to diminish apoptosis, which demonstrated the therapeutic potential of IRE1α signaling cascade as a target.

**Figure 7:**
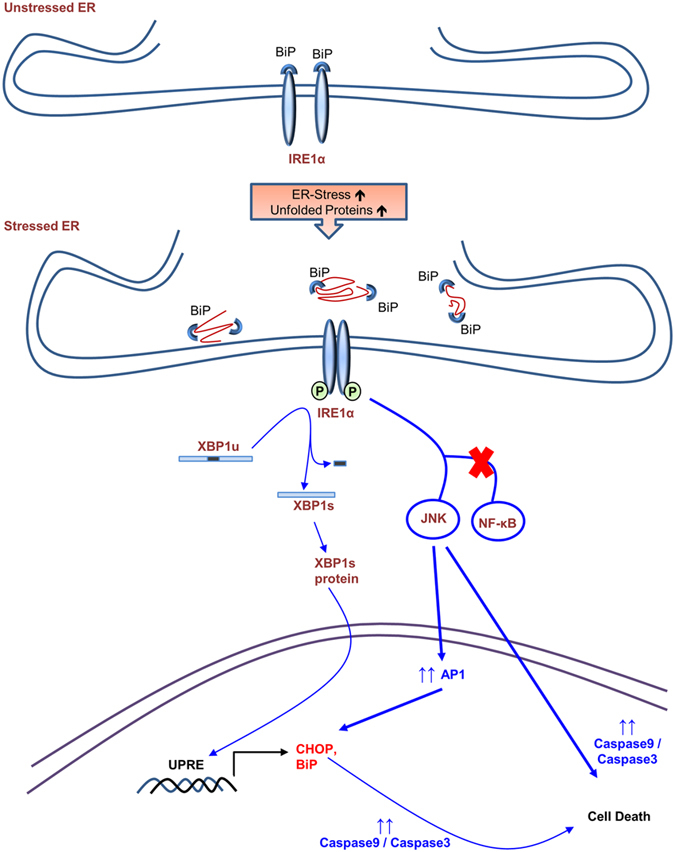
Schematic of signaling pathways involved in HIV-1 gp120-mediated ER stress astrocytes. The major signaling pathway sought in the present work is IRE1α signaling cascade, which is responsible for HIV-1 gp120-mediated ER stress. The increase in BiP and CHOP levels suggest increased ER stress. This activates IRE1α pathway, which further increases splicing of XBP-1 and activation of JNK. The post-translational activation of JNK further activates AP-1, which functions as a transcription factor to increase the expressions of CHOP. The increase in ER stress in this manner then activates the intrinsic caspase cascade via caspase-3 and caspase-9 cleavage. Together, these cascades ultimately lead to cell death in astrocytes.

The apoptotic potential of HIV-1 gp120 has been well documented in a variety of cells in the CNS including astrocytes, which includes both the intrinsic and the extrinsic pathways^4^,^42^,^43^,^44^. Our findings in the current study are consistent with earlier reports. Furthermore, the alteration of calcium levels in the ER is largely associated with ER stress during several neurological disorders. A prior study form Haughey *et al.* demonstrated that HIV-1 gp120 dysregulated calcium levels in neurons, suggesting a possible link between HIV-1 gp120 and ER stress^42^. More recently, brain studies of HIV patients demonstrated increased ER stress markers in various cells in CNS including neurons and astrocytes^22^. Therefore, we hypothesized that gp120 could be responsible for the observed increase in ER stress. Our study aimed to demonstrate detailed mechanism underlying the HIV-1 gp120-mediated ER stress, which may further lead to apoptosis. Our results demonstrating time-dependent increase of BiP and CHOP expressions indicated that the ER stress induced via HIV-1 gp120 could persist over a longer duration. Under physiological conditions, the accumulation of unfolded proteins prompts the levels of BiP, which chaperones the unfolded proteins for ubiquitination^11^,^33^ to restore homeostasis. However, persistent ER stress activates the stress response beyond repair. In the present study, we reported increase in the levels of IRE1α as a result of HIV-1 gp120 exposure to astrocytes (Fig. 2). The activated IRE1α can further activate JNK via ASK1, which in turn activates transcription factor, AP-1^45^,^46^ (Fig. 7). Our results are consistent with this notion since we demonstrated increase in the phosphorylation of JNK, which then increased the levels of AP-1. Furthermore, we reported HIV-1 gp120-mediated splicing of XBP-1, which is a key step in the production of molecules that are responsible for folding and processing of the proteins in the ER. The frame-shift during the splicing of XBP-1 results in shorter XBP-1 protein, which increases transcription of downstream genes^36^,^47^ (Figs 3 and 7). A recent study suggested that blocking XBP-1 splicing could be a potential therapeutic target for multiple myeloma^48^. Thus, increased splicing of XBP-1 observed in the present study can be targeted for therapeutic intervention.

In addition to *in-vitro* results from astrocyte cell line and primary human fetal astrocytes, our results in the mouse brains from gp120 tg mice also indicated that HIV-1 gp120 increased ER stress in various regions of the brain. Our results clearly demonstrated an increase of all the molecules in the IRE1α pathway in the prefrontal cortex (PFC). In addition, BiP and CHOP were increased in the parietal cortex. Interestingly, PFC is involved in complex cognitive behavior and decision-making process. Thus, damage to PFC is directly associated with cognitive impairment as observed in HAND. Previously, HAND patients were reported to exhibit increased DNA damage in the frontal cortex^49^. Similarly, exposure of parietal cortex to HIV-1 gp120 increased the excitatory neurotransmitter, which may lead to transient neurological and psychiatric symptoms observed in HIV associated dementia^50^. Furthermore, gp120 tg mice demonstrated heightened damage to the dendritic cells in PFC^51^. The results presented herein provide further insight towards understanding the region specific damage induced by HIV-1 gp120.

The pro-survival or pro-apoptotic fate of the cells relies upon the extent and the length of the stress during ER stress. However, ER stress-mediated cell death is largely attributed to either IRE1α pathway or PERK pathway^34^. Likewise, various molecules such as JNK, CHOP and activation of caspase cascade are known to be responsible for cell death during ER stress^12^,^52^. This has also been observed in Aβ-induced ER stress, where inhibition of JNK activation was reported to trigger pro-survival response^53^. Similarly, increased levels of CHOP has also been reported to be associated with death in different cells in various disorders such as diabetes, brain ischemia and neurodegenerative disease^54^,^55^,^56^,^57^. This is evident by the fact that CHOP overexpression induces cell cycle arrest and apoptosis^54^,^55^ and its silencing reduces ER stress-mediated apoptosis^56^,^57^. Our findings clearly demonstrate that HIV-1 gp120-mediated ER stress induced astrocyte death, and silencing of any intermediary molecules partially protected them from death. The knockdown of BiP on the other hand increased cell death assessed via PI staining (statistical significance; p-value < 0.001). This is not surprising since BiP is essential for proper folding of the proteins^13^,^18^. Accumulation of the unfolded proteins in the event of BiP knockdown would exacerbate the stress in the ER lumen, which in turn could trigger cytotoxicity. Although it still remains to be determined whether PERK pathway plays an additional role in the HIV-1 gp120-mediated cell death, the results in the present study provides a potential target for therapeutic intervention for prevention of the HIV-1 gp120-mediated cell death.

Caspase cascade is a classical mechanism responsible for pro-apoptotic response in various types of cells^58^. Depending on the activation of caspase cascade, the pathway could be either an extrinsic (primarily associated with extracellular stimulus via ligand binding to the death receptors such as TNF receptor-1; TNFR1 and FAS-associated death domain; FADD) or intrinsic (primarily associated with mitochondrial dysregulations due to a variety of stress including ER stress) pathway^58^. Particularly, caspase-8 largely accounts for extrinsic apoptotic events under death receptor-mediated activation while the caspase-3, 6, 7 and 9 are associated with the intrinsic pathway. In the present study, we did not observe alteration in the expression or activation of caspase-8 after HIV-1 gp120 exposure (data not shown). However, the levels of cleaved caspase-3 and -9 were significantly higher in HIV-1 gp120 exposed astrocytes (Fig. 6). The cleaved caspase-3 and -9 increase was partially abrogated after silencing of any of the IRE1α intermediates. This was further confirmed as pan-caspase inhibitor, ZVAD-FMK reduced HIV-1 gp120-mediated apoptosis. Thus, these results indicate that HIV-1 gp120-mediated ER stress activated the intrinsic caspase pathway, which could eventually lead to cell death in astrocytes. This is in agreement with our previous report, where we demonstrated that HIV-1 gp120-mediated oxidative stress resulted into cell death via activation of caspase-3^4^. Furthermore, we have demonstrated that HIV-1 gp120 activates NFκB to induce the production of various pro-inflammatory cytokines and chemokines^5^,^6^,^7^. Therefore, our present study provides further insight into the intricate mechanisms that ultimately lead to cell death via various routes.

In summary, the results from our present study provide a novel insight into the possible involvement of ER stress in HIV-1 gp120-mediated cytotoxicity in astrocytes. In addition, the involvement of IRE1α pathway in particular presents an opportunity to explore possible targets for the therapeutic intervention or to further understand the complicated mechanisms underlying HIV-1 gp120-mediated neurotoxicity. In future, various synthetic or natural compounds can be tested to reduce the HIV-1 gp120-mediated ER stress and potentially reduce the HIV-1-associated neuropathogenesis. Various natural compounds such as quercetin and sulforaphane possess antioxidant properties and have been employed to reduce ER stress^59^,^60^,^61^. These natural compounds can be tested to further evaluate their potential in the mitigation of HIV-1 gp120-mediated ER stress.

## Methods

### Cells and reagents

All the *in vitro* experiments were carried out with either SVGA cells, a human astrocyte cell-line unless specified or primary cultures of human fetal astrocytes (HFA). HFA were prepared from aborted fetal tissues obtained from Birth Defect Research Laboratory (BDRL), Seattle, WA. The cells were characterized by the expression of GFAP and >98% cells were found to be positive (data not shown). For experiments with SVGA cells, 2.5 × 10^5^ cells were cultured in 12-well plates overnight for adherence followed by transfection with a plasmid expressing HIV-1 gp120 (pSyngp120-JRFL; catalog # 4598, NIH AIDS Reagent Program) for various durations. The cells were harvested and further processed for either RNA isolation or protein preparation. Similarly, for HFA, 1 × 10^6^ cells were treated with different concentrations of recombinant HIV-1 gp120 protein (gp120 IIIB CHO; Catalog # 11784, NIH AIDS Reagent Program) for various time lengths. All the cells were grown in complete DMEM medium containing (1% L-glutamine, 1% sodium bicarbonate, 1% non-essential amino acids, 10% fetal bovine serum and 25 mg gentamicin sulfate) at 37 °C in a humidified chamber containing 5% CO_2_. Specific siRNA (ON-TARGETplus SMART pool) for BiP, CHOP, IRE1α, JNK, AP-1, p65, and control siRNA were purchased from Dhramacon (Thermo Fisher Scientific Inc., Waltham, MA, USA). Specific antibody against p-JNK was purchased from Santa Cruz Biotechnology (Santa Cruz, CA, USA), glyceraldehyde 3-phosphate dehydrogenase (GAPDH) (FL-335), caspase-3, which detects procaspase-3 (35  kDa) and cleaved caspase-3 (17 kDa), caspase-9 for both procaspase-9 (47 kDa) and cleaved caspase-9 (35 kDa), IRE1α, CHOP and BiP were purchased from Cell Signaling Technology (Danvers, MA, USA) and antibody against XBP-1 and phospho IRE1α were purchased from Abcam (Cambridge, MA, USA). Irreversible pan-caspase inhibitor, ZVAD-FMK was purchased from Apex Bio (Boston MA, USA).

### Animals

The present study was performed using 3–4 month old 12 male SJL mice (6 WT control and 6 gp120 transgenic), which were originally generated as previously described^62^. The mice contained a genetic background of SJL/BL6/Sv129 expressing CCR5WT (Originally BL6xSv129) and heterozygous expression of gp120 allele under the regulatory control of modified murine glial fibrillary acidic protein (GFAP). These mice were obtained from Dr. Marcus Kaul at Sanford Burnham Medical Research Institute and bred in the UMKC-Laboratory Animal Research Core (LARC) facility. All the mice in the study were housed in a group of 3–5 animals per cage in a controlled environment with 12 h light/dark cycle (lights off at 7:00 AM) and *ad libitum* access to food and water. This study was in accordance with the NIH guidelines and the experimental protocols were approved by the institutional animal care and use committee (IACUC) at UMKC.

### Transfection

SVGA cells were transfected as mentioned previously^4^,^5^,^6^,^7^. Briefly, monolayer of SVGA cells in 6 or 12-well plates were transfected with HIV-1 gp120 plasmid using Lipofectamine2000^TM^ (Life Technologies, Carlsbad, CA, USA) for 5 hours followed by replacement of the transfection cocktail with complete DMEM medium containing serum. Cells transfected with empty vector were used as controls in all the transfection experiments.

For the experiments involving siRNA transfections, 6 × 10^5^ cells were transfected with 20 nM of siRNA in a 6-well plate. Briefly, the cells were washed twice with PBS to remove serum and incubated with serum-free growth medium containing transfection cocktail. After 24 hours, the growth medium containing transfection cocktail was replaced with complete medium containing serum for additional 10 hours. The cells were then recounted and seeded at 2.5 × 10^5^ cells per well in a 12-well plate. These gene-silenced cells were further transfected with gp120 to assess the effect of specific target knockdown. The efficiency of gene silencing was confirmed using western blotting.

### Real-time reverse transcriptase-polymerase chain reaction

To measure the mRNA expression levels of BiP and CHOP, astrocytes were exposed to HIV-1 gp120 for various durations. Upon termination of the treatment, total RNA was isolated using Qiagen RNeasy Mini Kit (Qiagen, Valencia, CA, USA). The RNA (150 ng) was reverse transcribed at 37 °C for 60 min followed by amplification of the target mRNA. The expression of BiP was measured using forward primer: 5′-CGAGGAGGAGGACAAGAAGG-3′ and reverse primer: 5′-AGTTCTTGCCGTTCAAGGTG-3′ and amplification conditions (Annealing at 62 °C for 30 sec, denaturation at 95 °C for 15 sec). Similarly, CHOP was measured using forward primer: 5′ GCACCTCCCAGAGCCCTCACTCTCC-3′ and reverse primer: 5′-CGCAGGGGGAAGGCTTGGAGTAGAC-3′ using amplification conditions (Annealing at 62 °C for 30 sec, denaturation at 95 °C for 15 sec). The expression values were normalized using Hypoxanthine-guanine phosphoribosyltransferase *(HPRT)* as a housekeeping gene. Relative fold expressions for various genes were analyzed using the 2^−ΔΔCt^ method.

### Determination of spliced XBP-1 using agarose gel

Total RNA were isolated from astrocytes and 150 ng RNA template was reverse transcribed and amplified using XBP-1 primers (Forward: 5′-TTACGAGAGAAAACTCATGGCC-3′ and reverse: 5′-GCATTCTGGACAACTTGGACCC-3′) that amplifies spliced XBP-1 (263 bp) and unspliced XBP-1 (289 bp) using optimized PCR conditions (Annealing at 62 °C for 30 sec, denaturation at 95 °C for 15 sec). The amplified PCR product was mixed with loading dye followed by electrophoresis at 120 V for 90 min using 3.5% agarose gel prepared in TBE buffer with ethidium bromide. The desired RNA bands were visualized using gel documentation camera combined with FluorChem HD2 software (Alpha Innotech, San Leandro, CA, USA) and the band intensities were normalized using HPRT as loading control.

### Western blotting

The expressions of various signaling molecules were measured in whole cell lysates as described previously^6^. Briefly, the whole cell lysates were prepared by incubation with radioimmunoprecipitation assay (RIPA) buffer (Boston BioProducts, Ashland, MA, USA) supplemented with Halt^TM^ protease inhibitor cocktail (Thermo Fisher Scientific Inc., Waltham, MA, USA) for 10 min at 4 °C followed by homogenization for 30 sec. Cell debris was removed by centrifugation of the lysates at 14000 Xg for 10 min at 4 °C. The supernatants were stored at −80 °C until use.

The protein concentrations were quantified using BCA protein assay kit (Thermo Fisher Scientific Inc., Waltham, MA, USA) and 20 μg protein was resolved using 10–12% SDS-polyacrylamide gel electrophoresis at 80 V for 2 hours. The resolved proteins were transferred onto a PVDF membrane with constant 350 mA current for 75 min and the membrane was blocked with 5% nonfat milk in PBST (0.075% Tween 20 in PBS) overnight at 4 °C to reduce nonspecific signals. The membrane was then incubated with optimized concentrations of primary antibody to probe for target proteins for 2 hours at room temperature, washed 5X with PBST and again probed with respective horse radish peroxidase conjugated secondary antibody for 2 hours at room temperature. Various target protein bands were visualized using BM chemiluminescence western blotting substrate (POD, Roche Applied Sciences; Indianapolis, IN, USA) and quantified with FluorChem HD2 software (Alpha Innotech, San Leandro, CA, USA). All western blotting were performed with same experimental conditions and the loading controls were run for each blot individually.

### Cell survival (MTT assay)

The cells were seeded in a 12-well plate at the density of 2 × 10^5^ cells/well and appropriate treatments were performed. Cell viability was determined using the colorimetric 3-(4,5-dimethylthiazol-2-yl)-2,5-diphenyltetrazolium bromide (MTT) reagent at 48 hours, which is converted into purple formazan crystals by mitochondrial dehydrogenase in the viable cells. Briefly, the cells were treated with 0.2 mg/ml MTT solution prepared in serum-free medium and incubated at 37 °C for 3–4 hours. The process was terminated by carefully removing the MTT reagent and lysing the cells with 500 μl dimethylsulfoxide, which dissolves the formazan crystals to form a clear purple solution. The color intensity was measured using Benchmark Microplate Reader (Bio-Rad Laboratories, Hercules, CA, USA) with absorbance at 570 nm and reference at 650 nm. Cell viability was calculated with the absorbance in control cells as 100%.

### Propidium Iodide (PI) staining for cell viability

The cells were seeded in 12-wel plate at 2 × 10^5^ cells/well and transfected with HIV-1 gp120 plasmid for 48 hours. Upon termination of the experiment, the cells were trypsinized and suspended in ice cold PBS. The cell culture supernatants were also pooled together to ensure the recovery of the dead cells in the suspension. After 2 washes in PBS, the cells were incubated with PI solution in PBS at 1 μg/ml concentration for 15 min in dark. The fluorescence was immediately measured using flow cytometer and percent dead cells were reported as absolute values by gating the background fluorescence in unstained cells. The effect of various siRNA-mediated knockdown of the respective targets was measured using similar approach with an additional siRNA transfection as mentioned in transfection section in Methods.

## Statistical Analysis

The statistical analysis was performed to represent the data in mean ± S.E. values. Results were based on at least three separate experiments unless specified, with each experiment performed in triplicates. For the comparison between mock/control group and treatments two-tailed Student’s t-test was applied to calculate P-values, and P-value ≤ 0.05 was considered statistically significant. The P-value ≤ 0.05 was indicated by ‘*’ and ≤0.01 was indicated by ‘**’ in the bar graphs. Experiments involving multiple variables such as an inhibitor or siRNA were analyzed using one-way ANOVA with multiple comparisons to address whether the suppression is significant. The western blots shown in each panel is a representative blot with each experiment performed at least 3 times. The mean ± S.E. are presented in the bar graphs. No adjustments were made in the statistical analysis for multiple comparisons.

## Additional Information

**How to cite this article**: Shah, A. *et al.* HIV-1 gp120 induces type-1 programmed cell death through ER stress employing IRE1α, JNK and AP-1 pathway. *Sci. Rep.* **6**, 18929; doi: 10.1038/srep18929 (2016).

## Supplementary Material

Supplementary Figure 1
