# Supplementary material for: HIV-1 gp120 induces type-1 programmed cell death through ER stress employing IRE1α, JNK and AP-1 pathway
Source: Sci Rep. 2016 Jan 7;6:18929. doi: 10.1038/srep18929 (PMC4703964; doi:10.1038/srep18929)
Supplement: Supplementary Figure 1 [file srep18929-s1.pdf]

HIV-1 gp120 induces type-1 programmed cell death through ER stress employing IRE1α, JNK and AP-1 pathway

Ankit Shah[1], Naveen K. Vaidya[2], Hari K. Bhat[1], Anil Kumar[1]
[1]Division of Pharmacology and Toxicology, School of Pharmacy, [2]Department of Mathematics and Statistics, University of Missouri-Kansas City, Kansas City, MO 64108

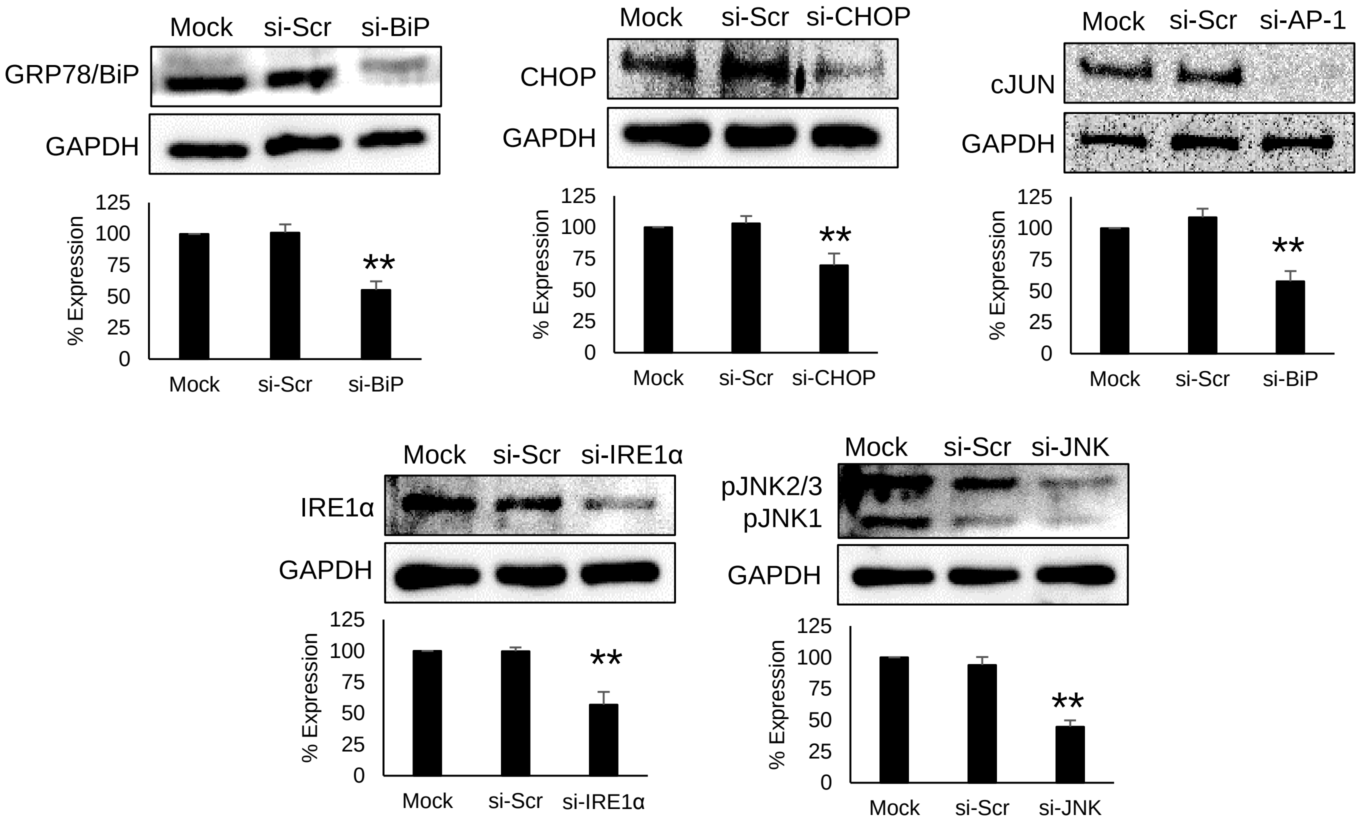

Supplementary Fig. 1 : The efficiency of siRNA-mediated depletion of the target molecules: SVGA cells were transfected with 20 nM of siRNA in a 6-well plate as described in Methods section. After 48 hours, the cells were lysed with lysis buffer and the protein levels were determined with western blotting. The bar graphs shown in the figure are represented in mean ± S.E., while the western blots are representative images. The blots presented in the figures were obtained by cutting membranes at the molecular markers above and below protein of interest before probing them for appropriate primary and secondary antibodies. The images are then presented as is with brightness/contrast adjustment applied throughout the blot without altering the overall results. The statistical significance was calculated using one-way ANOVA with multiple comparisons and the values were considered significant if p-value ≤ 0.05 (*) or ≤ 0.01 (**).
